# TGF-β1 is a regulator of the pyruvate dehydrogenase complex in fibroblasts

**DOI:** 10.1038/s41598-020-74919-8

**Published:** 2020-10-21

**Authors:** Edward R. Smith, Timothy D. Hewitson

**Affiliations:** 1 Department of Nephrology, The Royal Melbourne Hospital (RMH), Grattan Street, Parkville, VIC 3050 Australia; 2 Department of Medicine – RMH, University of Melbourne, Parkville, VIC Australia

**Keywords:** Cell biology, Nephrology

## Abstract

TGF-β1 reprograms metabolism in renal fibroblasts, inducing a switch from oxidative phosphorylation to aerobic glycolysis. However, molecular events underpinning this are unknown. Here we identify that TGF-β1 downregulates acetyl-CoA biosynthesis via regulation of the pyruvate dehydrogenase complex (PDC). Flow cytometry showed that TGF-β1 reduced the PDC subunit PDH-E1α in fibroblasts derived from injured, but not normal kidneys. An increase in expression of PDH kinase 1 (PDK1), and reduction in the phosphatase PDP1, were commensurate with net phosphorylation and inactivation of PDC. Over-expression of mutant PDH-E1α, resistant to phosphorylation, ameliorated effects of TGF-β1, while inhibition of PDC activity with CPI-613 was sufficient to induce αSMA and pro-collagen I expression, markers of myofibroblast differentiation and fibroblast activation. The effect of TGF-β1 on PDC activity, acetyl-CoA, αSMA and pro-collagen I was also ameliorated by sodium dichloroacetate, a small molecule inhibitor of PDK. A reduction in acetyl-CoA, and therefore acetylation substrate, also resulted in a generalised loss of protein acetylation with TGF-β1. In conclusion, TGF-β1 in part regulates fibroblast activation via effects on PDC activity.

## Introduction

Chronic Kidney Disease (CKD) is characterized by non-recoverable organ remodelling and fibrosis. Histologically this process consists of glomerular and vascular sclerosis, and tubulointerstitial fibrosis, with the latter being the best predictor of progression in CKD^1^.

The interstitial fibroblast is a major effector cell in this process^2^. However, like fibroblasts elsewhere, they represent a heterogeneous population of cells which makes identification difficult. De novo expression of αsmooth muscle actin (αSMA) is often used to identify a sub-set of fibroblasts termed myofibroblasts^2^. While they only represent a proportion of collagen producing cells^3^, these cells are a prodigious source of collagen, and correlate with progression of fibrosis. αSMA expression is therefore often used as a surrogate marker of activation and aberrant fibrogenesis^2^.

Despite the importance of these cells, the mechanisms involved in their sustained activation and persistence are poorly understood. In cancer tumours it has long been recognised that changes in cellular metabolism help cells acquire and maintain a malignant phenotype^4^. More recently we have also seen that metabolic reprograming plays an essential role in the differentiation and cell fate commitment of pluripotent stem cells^5^. Likewise there is also mounting evidence of a reprogramming of cellular metabolism in various forms of fibrosis^6–8^, including myofibroblast-like cells^8,9^ in keloids^10^, hepatic^11,12^, lung^8^ and perioteneal^13^ fibrosis. Although TGF-β1 is a specific driver in many cases^11–13^, the molecular events underpinning these global changes remain ill-defined.

The initial differentiation of fibroblasts to myofibroblasts and maintenance of the activated state is a growth factor driven process^2^. Since the landmark studies of Border et al. demonstrated a role for transforming growth factor beta 1 (TGF-β1) in glomerulosclerosis^14^, a multiplicity of evidence has implicated TGF-β1 as the pre-eminent fibrogenic signal^15^. However, despite the established significance of TGF-β1 in fibrogenesis, its mechanism of action is still surprisingly poorly understood.

A serendipitous finding in our recent studies was that TGF-β1 induced a shift in metabolism from oxidative phosphorylation to aerobic glycolysis in fibroblasts, which was associated with a marked reduction in intracellular free acetyl-CoA concentration^16^. Under physiological conditions, more than 70% of acetyl-CoA is generated from pyruvate by the mitochondrial multienzyme pyruvate dehydrogenase complex (PDC)^17^. PDC occupies a critical position in the oxidation of glucose, as acting as the gatekeeper between glycolysis and the tricarboxylic acid (TCA) cycle. During myofibroblast differentiation, a switch in respiration from oxidative phosphorylation to aerobic glycolysis therefore implies a potential block in flux through PDC, and re-routing of carbon to support the high synthetic demands for de novo expression of extracellular matrix proteins (i.e. collagen) as well as cytoskeletal and contractile apparatus.

Activity of the complex is tightly regulated by covalent post-translational modifications and is principally achieved through the reversible phosphorylation of one of 3 regulatory sites in the E1a subunit which results in inactivation. Phosphorylation of PDC is dependent on the opposing actions of pyruvate dehydrogenase kinases (PDK)1–4 and pyruvate dehydrogenase phosphatases (PDP) 1 and 2, which are themselves regulated at both transcriptional and post-transcriptional levels by a number of metabolic inputs^18^.

Metabolic reprogramming may also have more widespread implications^19^. Not only is acetyl-CoA a substrate for the TCA cycle and other biosynthetic pathways, it is also the obligatory acetyl donor for regulatory lysine acetylation^20^, thus providing a direct link between alterations in cellular metabolism and protein function. Although phosphorylation arguably represents the best understood protein modification, more recent attention has focused on acetylation, in large part due to interest in the role of histone acetylation in epigenetic regulation of transcription. However, the significance of protein acetylation extends well beyond the regulation of histones and gene expression^21,22^ with proteomic analysis showing that more than 2,000 non-histone proteins are acetylated in the kidney^23^, some with very rapid rates of turnover^24,25^. Nevertheless, the coupling of TGF-β1 signalling, metabolic reprogramming and broader downstream effects on protein acetylation has yet to be proven.

Here we investigate the key metabolic events that are necessary for initiation and maintenance of fibroblast activation and explore their downstream consequences for protein acetylation. This study reveals that TGF-β1 downregulates acetyl-CoA biosynthesis via coordinated changes in the transcriptional and post-translational regulation of PDC, leading to a global reduction in protein acetylation.

## Results

### TGF-β1 modifies acetyl-CoA biosynthetic pathways in renal fibroblasts

**Figure 1:**
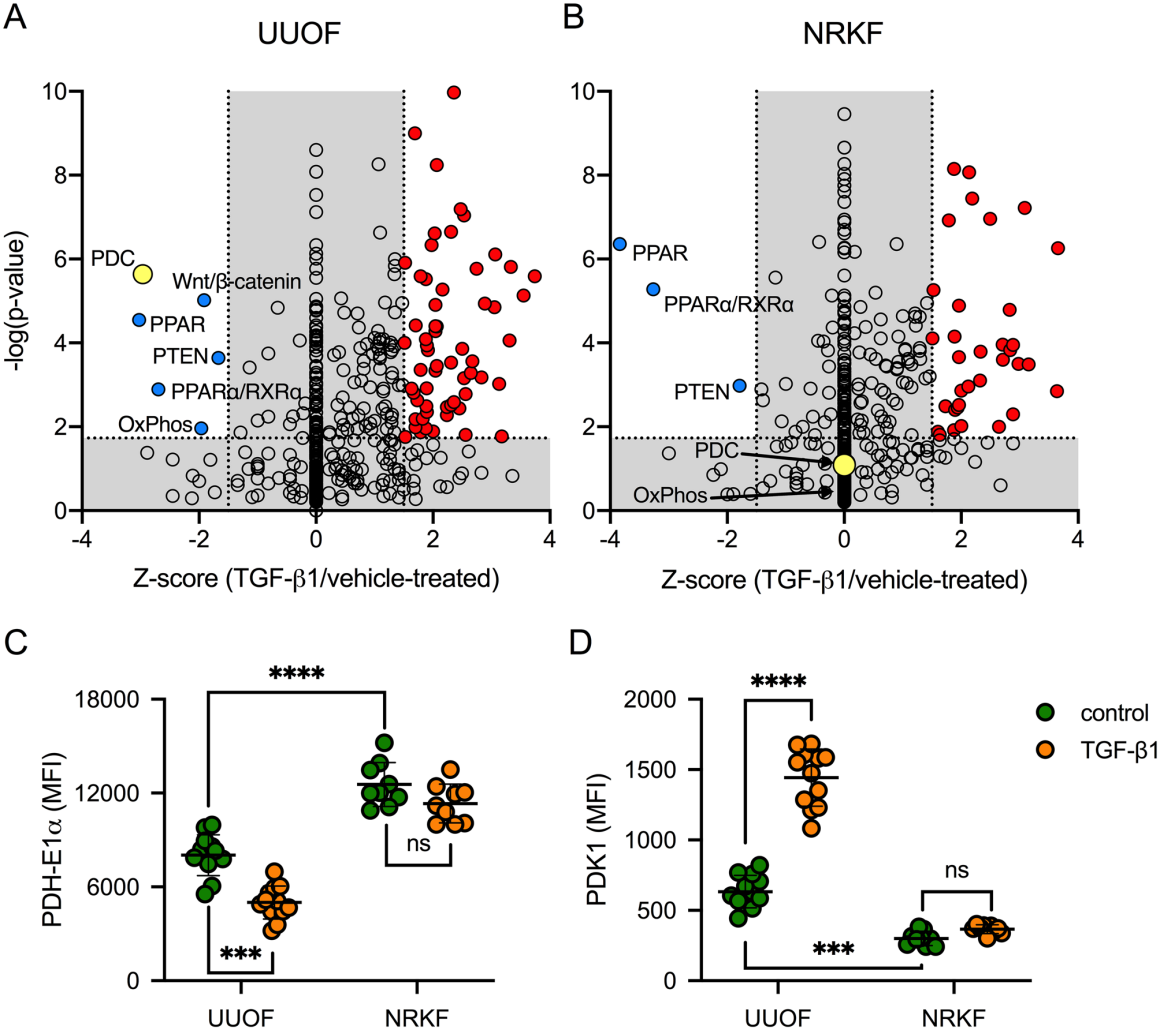
Divergent effects of TGF-β1 on PDC transcription in injury-primed and normal renal kidney fibroblasts. (**A**, **B**) Ingenuity pathway analysis (IPA) was used to predict the activation state of canonical pathways activated or inhibited by 24-h treatment with 1 ng/mL TGF- β1 compared to vehicle (PBS) in (**A**) UUOF or (**B**) NRKF. IPA uses a literature-based database of known interactions between transcriptional regulators and their target genes to define an expected expression pattern of targets within each pathway for activated (Z-score ≥ 2) or inhibited (Z-score ≤ − 2) states. The P-value provides a measure of how likely the observed association between a specific pathway and each dataset would be if due to chance alone. Adjusted P-value < 0.05 (− log_10_ = 1.3) were considered significant. Canonical pathways predicted to be activated by TGF-β1 are shown in red, inhibited pathways are coloured blue with the remainder depicted as open circles. The acetyl-CoA biosynthesis (PDC) pathway is highlighted in yellow. (**C**, **D**) Quantitative flow cytometric analysis of TGF-β1-induced changes in (**C**) PDH-E1α and (**D**) PDK1 staining (mean fluorescence intensity, MFI) in UUOF and NRKF after 24 h treatment (pooled data from 3 independent experiments). Plots show mean ± SEM. ***P < 0.001; ****P < 0.0001; ns, not significant. P-values were determined in (**C**, **D**) using two-way ANOVA with Sidak’s multiple comparisons test.

To better understand the pathways involved in driving the metabolic effects in response to TGF-β1, we performed an Ingenuity Pathway Analysis (Qiagen, Hilden, Germany) of our previously published RNA-seq database^16^ to predict the canonical pathways differentially regulated. In the first instance we analysed the whole transcriptome of fibroblasts propagated from rat kidneys 3 days after unilateral ureteric obstruction (UUOF), a model of primary tubulointerstitial fibrosis. Of the pathways predicted to be differentially regulated by TGF-β1 vs. vehicle, 63 were upregulated, while only 6 were inhibited (Fig. 1A). With respect to effects on metabolic pathways, both acetyl-CoA biosynthesis (PDC) and oxidative phosphorylation were significantly downregulated (Fig. 1A). mRNA transcripts for multiple subunits of the PDC^26^ were decreased by TGF-β1; *Pdha1* (fold change − 4.27, q value 0.004), *Pdhb* (− 2.08, 0.007), *Dlat* (− 1.51, 0.0002) and *Dld* (− 1.97, 0.0004)^16^. The same analysis was also performed using fibroblasts propagated from normal rat kidney tissue (NRKF). Interestingly while the pattern of transcriptome changes was superficially similar, it was notable that TGF-β1 had no predicted effect on either PDC or oxidative phosphorylation canonical pathways in this cell population (Fig. 1B), suggesting that fibrotic cells are primed to respond differently to their normal counterparts.

In order to confirm these transcriptional changes at the protein level we used flow cytometry to quantify protein expression of the PDC subunit pyruvate dehydrogenase- E1α (PDH-E1α) in cells treated with and without TGF-β1 (Fig. 1C). Levels of PDH-E1α were lower in UUOF than in NRKF basally, and further reduced (38%) in UUOF by treatment with TGF-β1. In contrast, TGF-β1 treatment had no effect on PDH-E1α staining in NRKF (Fig. 1D).

As PDK1 is a major regulator of PDC activity^27^, we sought to determine if TGF-β1 also has direct effects on PDK1 protein levels. Levels of PDK1 were higher in UUOF than NRKF, while TGF-β1 increased protein levels of PDK1 more in UUOF than their normal counterparts (Fig. 1D). Given, the unique effects on fibroblasts derived from injured kidneys, subsequent studies focused on these cells.

### TGF-β1 regulates PDC activity

**Figure 2:**
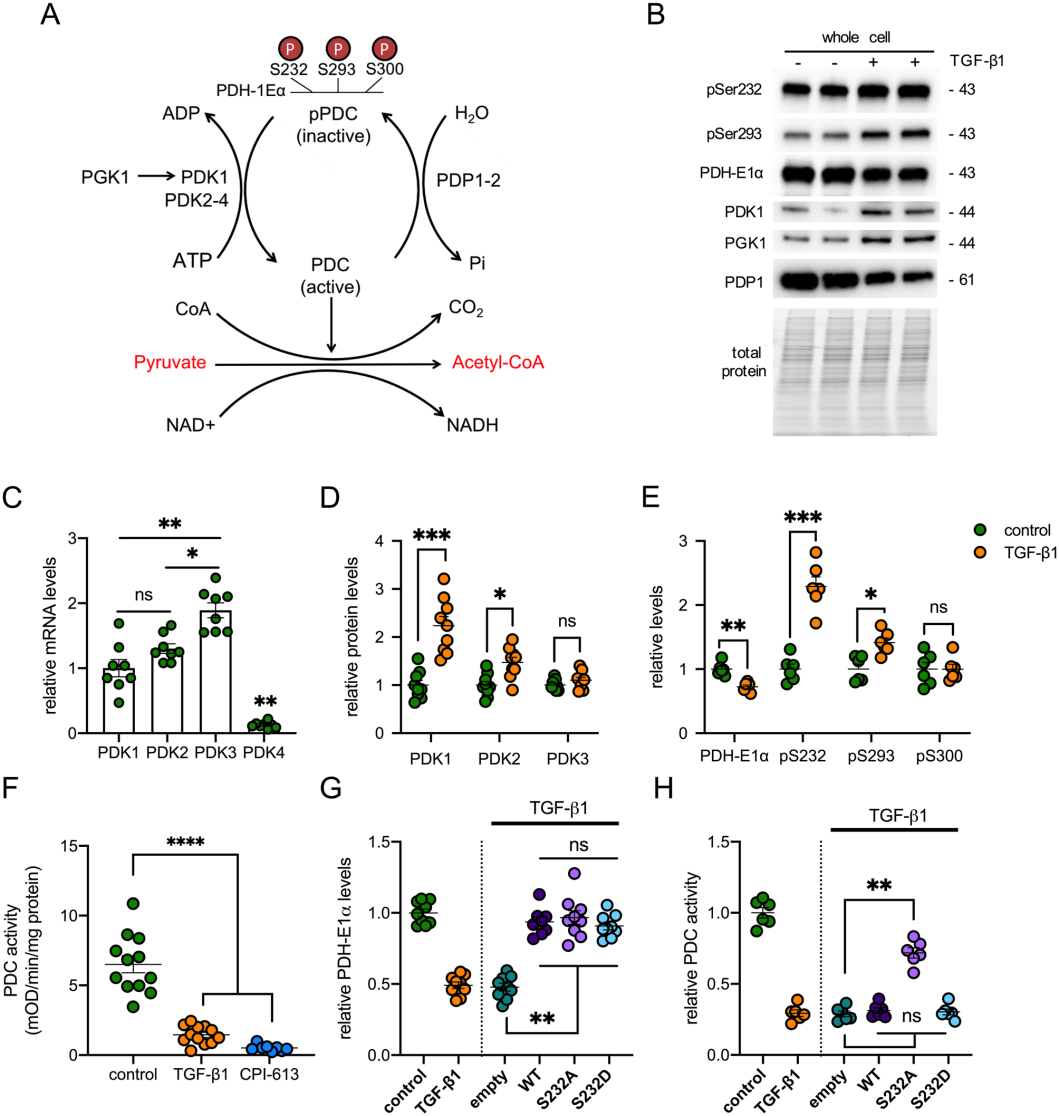
Effect of TGF-β1 on PDC activity and its regulation by phosphorylation. (**A**) Schematic showing the regulation of PDC activity by protein serine phosphorylation. (**B**) Western blots showing the effect of 1 ng/mL TGF-β1 for 24 h on phosphoserine 293, phosphoserine 232 and total PDH-E1α, PDK1, PGK1 and PDP1 in UUOF. Stain-free imaging of total protein was used to demonstrate equivalent loading. Representative blots of 3 independent experiments. (**C**) Relative abundance of mRNA transcripts for PDK1, PDK2, PDK3 and PDK4. Results are expressed relative to PDK1 levels and are pooled data from n = 3 experiments. (**D**) Flow cytometry measurement of protein levels of PDK1, PDK2 and PDK3 in control and TGF-β1-treated cells. (**E**) Bead-based multiplex assay of total PDH-E1α, phosphoserine (pS) 232, 293 and 300 with and without TGF-β1 treatment. Results in (**D**, **E**) are expressed relative to control and are pooled data from n = 3 independent experiments. (**F**) PDH enzyme activity in whole-cell lysates of vehicle, TGF-β1- and CPI-613-treated UUOF. Measurements were normalised to total protein content and pooled from n = 4 independent experiments. (**G**) PDH-E1α protein levels and (**H**) PDC activity in UUOF transiently transfected with plasmids over-expressing wild-type (WT) PDH-E1α or substitution mutants S232A and S232D. Empty vector was used as a control with values expressed relative to control levels and data pooled from n = 3 independent experiments. Plots show mean ± SEM. *P < 0.05; **P < 0.01; ***P < 0.001; ****P < 0.0001. P-values were determined using unpaired t tests and the two-stage method of Benjamini, Krieger and Yekutieli (FDR = 1%) in (**D**, **E**), and using Brown-Forsyth and Welch one-way ANOVA with Dunnett’s T3 multiple comparisons test in (**C**, **F**, **G**, **H**).

PDC is inactivated by reversible phosphorylation of the PDH-E1α subunit (Fig. 2A) at one of three serine residues (Ser232, Ser293, Ser300) through the site-specific actions of the PDK1-4 isoenzymes^18^. While all 4 PDKs are able to phosphorylate Ser293 and Ser300 residues, albeit at different rates, only PDK1 has activity at Ser232^28^. PDK1 is itself enzymatically activated by phosphoglycerate kinase 1 (PGK1)^29^. Dephosphorylation of PDH-E1α by phosphatases PDP1 and PDP2 in turn activates the complex^18^. In our study, Western blotting revealed that TGF-β1 increased phosphorylation of Ser232 PDH-E1α, which was paralleled by an increase in both PDK1 and PGK1, and a reduction in PDP1 (Fig. 2B; Supplementary Fig. S1). mRNA transcripts for all four PDK isoforms were detected using qRT-PCR, with PDK3 most abundant and PDK4 least abundant (Fig. 2C). Relative protein levels of PDK1 and PDK2 were increased by 220% and 50% respectively by TGF-β1, with no change in PDK3 (Fig. 2D). PDK4 protein was undetectable (data not shown). Of the three regulatory serine residues, phosphorylation of Ser232 was robustly increased by TGF-β1, with a more modest increment in Ser293 and no change at Ser300 (Fig. 2E). Consistent with these effects at the protein level, we found that TGF-β1 produced a marked 78% reduction in PDC activity, but quantitatively less than the inhibitory effect of CPI-613 (Fig. 2F), a non-redox active lipoate analog that stimulates phosphorylation of PDH-E1α through activation of PDKs^30^.

To corroborate the involvement of PDK1 and phosphorylation of Ser232 PDH-E1α in the regulation of PDC activity by TGF-β1, we compared the effect of over-expressing wild-type (WT) PDH-E1α and mutants in which Ser232 was mutated to alanine (S232A), to preclude phosphorylation, or to aspartate (S232D), to mimic permanent phosphorylation^31^. Transient transfection of TGF-β1-treated UUOF with plasmids encoding WT, S232A or S232D PDH-E1α restored PDH-E1α protein expression to control levels (Fig. 2G). However, consistent with inhibitory effects of TGF-β1 being specifically mediated by de novo phosphorylation of S232, only over-expression of the S232A PDH-E1α mutant yielded augmented PDC activity (Fig. 2H).

**Figure 3:**
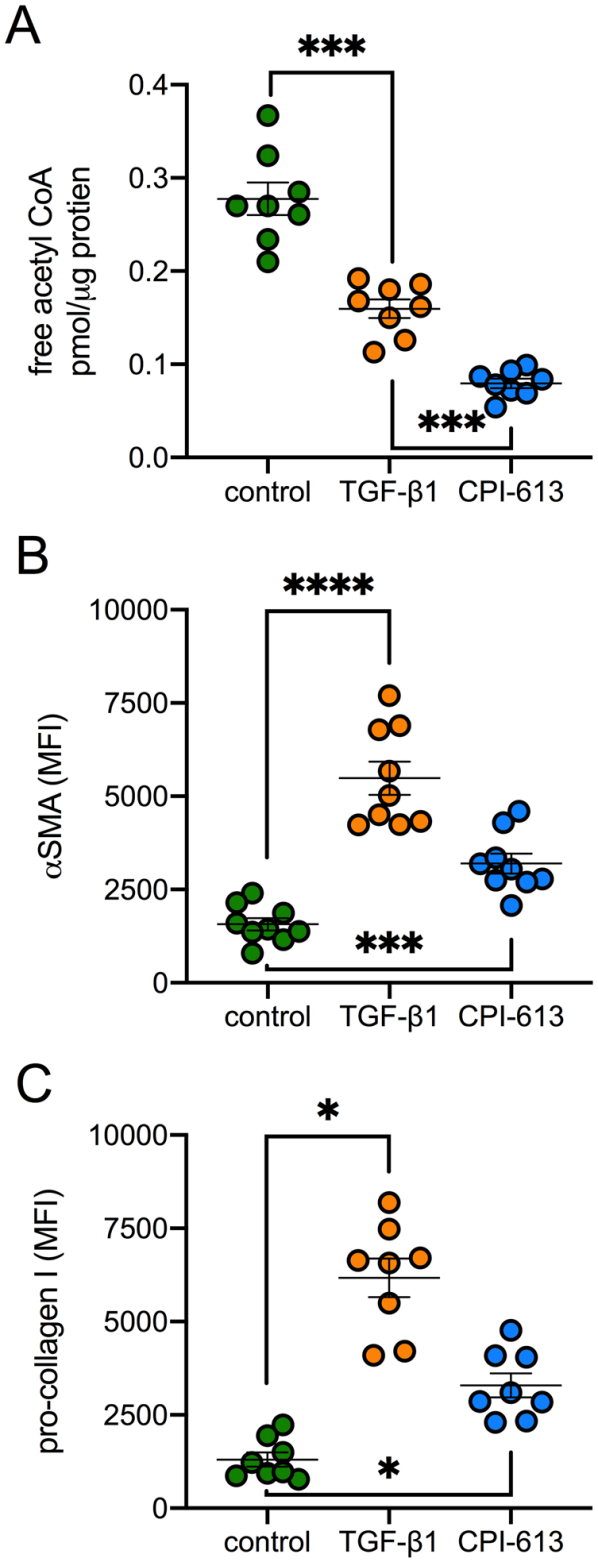
Effect of TGF-β1 on acetyl-CoA and fibroblast activation. (**A**) Enzymatic measurement of free acetyl-CoA concentrations (**B**), α-SMA and (**C**), pro-collagen I levels in UUOF treated for 24 h with TGF-β1 (1 ng/mL) or CPI-613 (100 µM) compared to control (pooled data from 3 independent experiments). Measurements in (**A**) were normalised for total protein content. Plots shows mean ± SEM. *P < 0.05; ***P < 0.001; ****P < 0.0001. P-values were determined using Brown-Forsyth and Welch one-way ANOVA with Dunnett’s T3 multiple comparisons test.

In agreement with PDC activity being a major contributor to cellular acetyl-CoA production, treatment with CPI-613 reduced acetyl-CoA levels by 71% (Fig. 3A). Surprisingly, however, CPI-613 treatment was also associated with an increase in αSMA (Fig. 3B) and pro-collagen I (Fig. 3C) levels, suggesting that inhibition of PDC alone is sufficient to enhance myofibroblast differentiation and fibrogenesis. As previously shown^16^, TGF-β1 also reduced acetyl-CoA levels (Fig. 3A), markedly increased αSMA levels (Fig. 3B) and as expected, increased pro-collagen I (Fig. 3C).

### Compartmentalisation of the PDC

**Figure 4:**
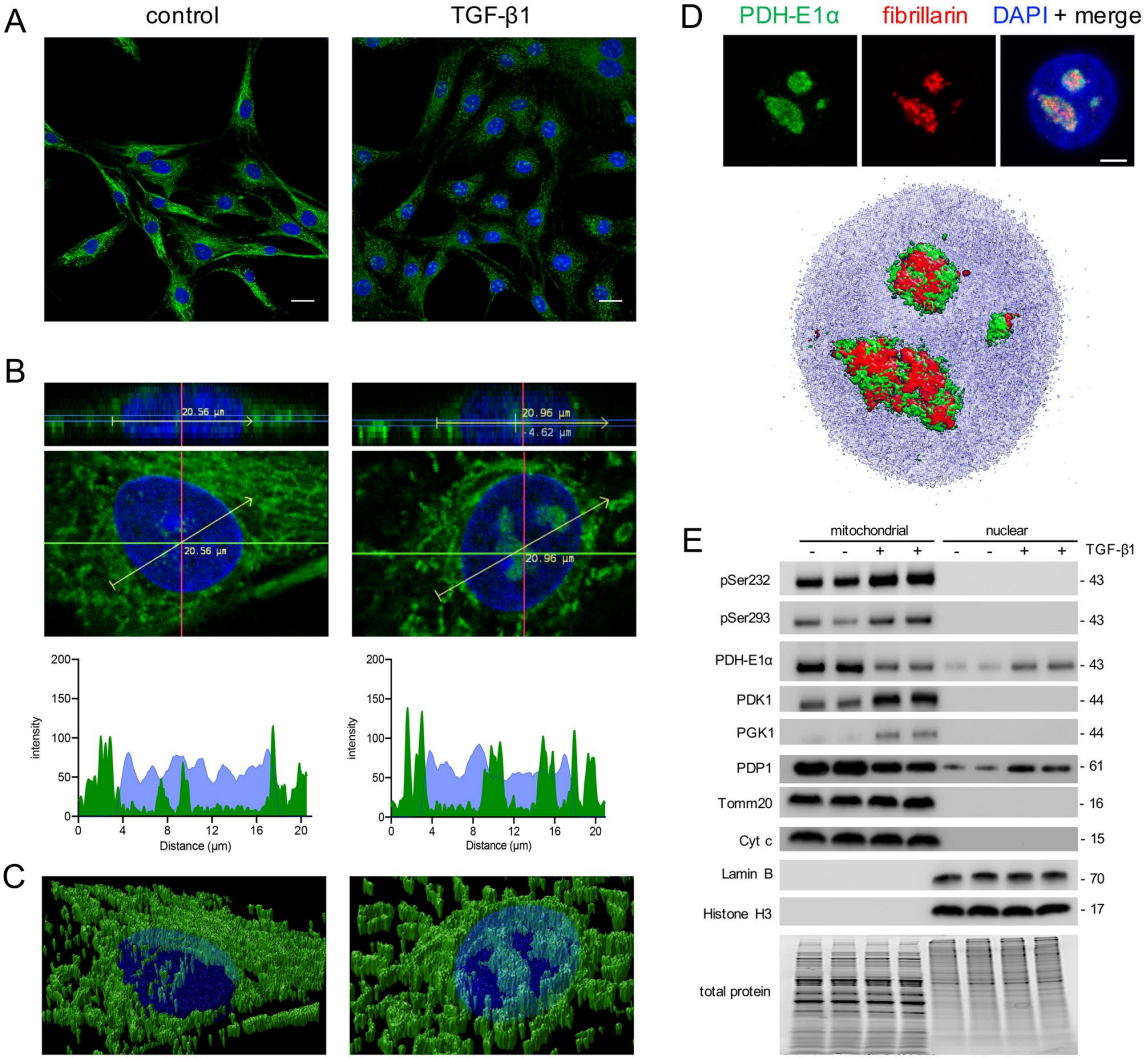
Effect of TGF-β1 on the compartmentalisation of PDH in UUOF. (**A**) Representative immunofluorescent staining of PDH-E1α (green) in UUOF with and without treatment with exogenous TGF-β1 (1 ng/mL) for 24 h. Nuclei were stained with DAPI (blue). Bar = 20 µm. (**B**) Representative high-power axial and frontal planes of nuclei from each experimental group. Line plots beneath show PDH-E1α (green) and DAPI (blue) staining intensity across a central nuclear plane (yellow arrow) using Fiji Image J. (**C**) Volume rendered images of cells in (**B**) showing change in nuclear and peri-nuclear distribution of PDH-E1α (green) in response to TGF-β1. (**D**) Nuclear co-localisation of PDH-E1α (green) and the nucleolar protein fibrillarin (red) after TGF-β1 treatment shown as individual stains, merged with DAPI (blue) and as a volume-rendered image. Bar = 5 µm. 3D rendered images were generated using Huygens Professional v19.04 (Scientific Volume Imaging; https://svi.nl). (**E**) Western blots showing the effect of 1 ng/mL TGF-β1 for 24 h on phosphoserine 293, phosphoserine 232 and total PDH-E1α, PDK1, PGK1 and PDP1 in mitochondrial and nuclear subcellular fractions of UUOF shown in Fig. 2B. Tomm20/Cyt C and lamin B/histone H3 were used to demonstrate purity of mitochondrial and nuclear proteins, respectively. Stain-free imaging of total protein was used to demonstrate equivalent loading. Representative blots of 3 independent experiments.

Given that others have shown that the PDC can redistribute within the cell in response to stimuli^32–34^, next we examined if the sub-cellular distribution of PDC changed in our cells with TGF-β1 (Fig. 4). In untreated cells, confocal microscopy showed that staining for PDH-E1α was consistent with a mitochondrial distribution (Fig. 4A), which we confirmed by colocalisation with MitoTracker deep red (Supplementary Fig. S2). While TGF-β1 treatment reduced cytoplasmic staining, as shown with our FC (Fig. 1C) and blotting analyses (Supplementary Figs. S1, S3), high power imaging showed what appeared to be a parallel increase in nuclear staining (Fig. 4B). To confirm this, we created z-stacks of individual nuclei, and analysed fluorescent intensity of PDH-E1α and DNA staining (DAPI) across a midline nuclear plane in control and TGF-β1-treated cultures (Fig. 4B). These cross-sectional analyses showed minimal nuclear PDH-E1α in the control group but increased nuclear staining in the TGF-β1-treated cells (Fig. 4B), which is also readily apparent in 3D reconstructions (Fig. 4C). Because of the very focal nuclear staining after TGF-β1 treatment, we considered that PDH-E1α might be localised to nucleoli. Double staining for PDH-E1α and the nucleolar marker fibrillarin confirmed their co-localisation (Fig. 4D).

Compartment specific changes in PDC regulation were also examined by Western blotting using isolated mitochondria and nuclear subcellular fractions (Fig. 4E). Purity of sub-fractions was confirmed by immunoblotting for the mitochondrial proteins Cyt C and Tomm20 and the nuclear proteins Lamin B and Histone H3. Consistent with the immunocytochemical analysis above, PDH-E1α was present in both mitochondria and nuclear fractions but was reduced by TGF-β1 in the mitochondria and increased in the nucleus (Fig. 4E; Supplementary Fig. S3). The presence of PDP1, but absence of both PDK1 and phosphorylated PDH-E1α in the nuclear fraction suggests that PDC may be maintained in an active state by PDP1 activity.

### Nuclear PDC is functional but is a minor source of cellular acetyl-CoA

**Figure 5:**
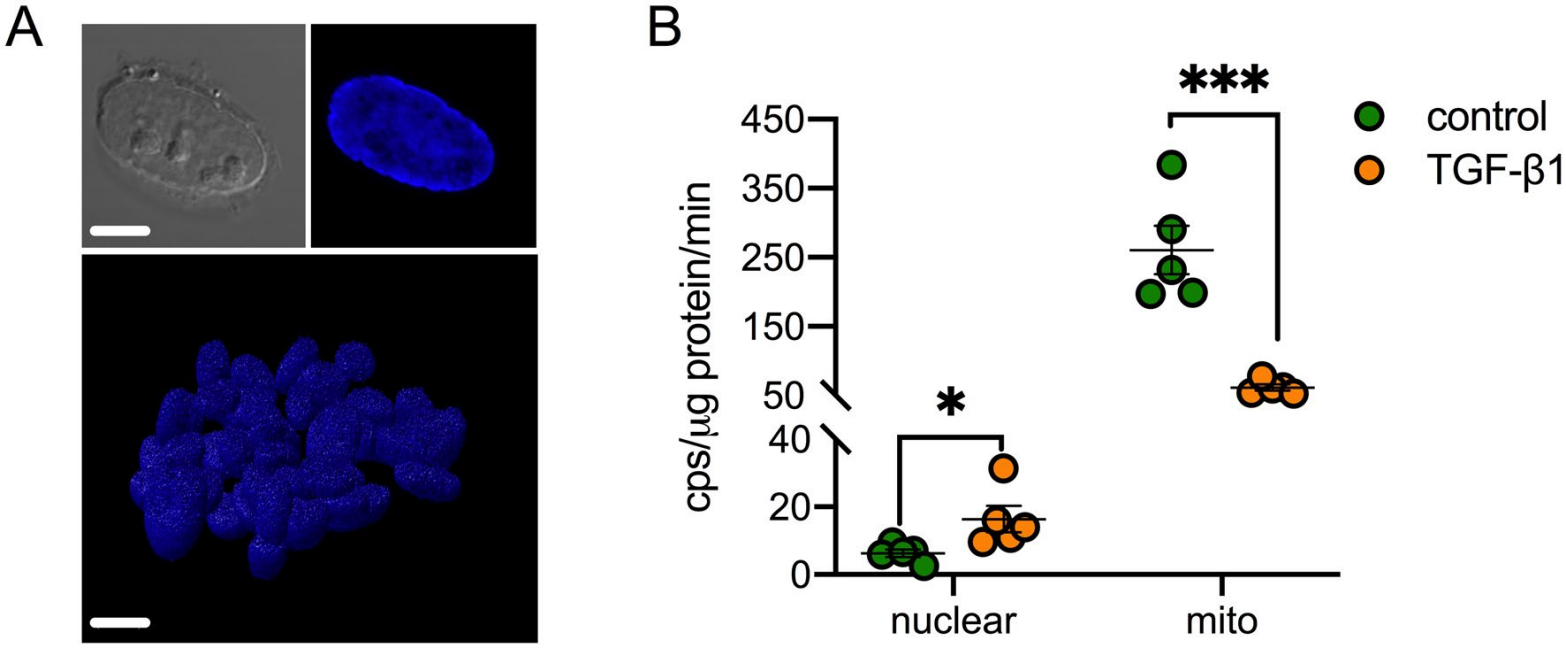
Nuclear PDC activity in response to TGF-β1. (**A**) Representative bright-field and fluorescent (DAPI) confocal images of intact nuclei isolated from UUOF. Top bar = 5 µm, bottom bar = 10 µm. (**B**) PDC activity in isolated nuclei and mitochondria with or without TGF-β1 (1 ng/mL) pre-treatment was determined by LC–MS/MS (cps, counts per second). Freshly prepared isolates of TGF-β1 or vehicle-treated UUOF were incubated with ^13^C_2_-pyruvate for 10 h at 37 °C after which metabolites were extracted for quantitation of ^13^C_1_-acetyl CoA. Nuclear PDC activity was higher in TGF-β1-treated UUOF compared to vehicle-treated cells, whereas mitochondrial PDC activity was lower in the TGF-β1-treated group compared to control isolates. Plot shows mean ± SEM of pooled data from 2 independent experiments (5 isolates in each experimental group). *P < 0.05; ***P < 0.001. P-values were determined in (**B**) using two-way ANOVA with Sidak’s multiple comparisons test.

To test whether nuclear PDC was active in fibroblasts, we first attempted to measure PDC activity in nuclear protein extracts using the same colorimetric kinetic assay applied to whole-cell activity measurements. Reaction rates, however, were similar to blank levels (data not shown) suggesting this approach was not sufficiently sensitive. As an alternative strategy, we next isolated intact nuclei from UUOF, and incubated these with isotope-labelled ^13^C_2_-pyruvate and measured de novo ^13^C_1_-acetyl-CoA synthesis by mass spectrometry (MS). Purity was confirmed by microscopy (Fig. 5A) and immunoblotting protein extracts for nuclear and mitochondrial specific markers (Fig. 4E). The validity of this approach was confirmed by first demonstrating that acetyl-CoA was generated in a protein (nuclei) and substrate dose-dependent manner and abrogated by of CPI-613 (Supplementary Fig. S4A–C respectively). Incubation of TGF-β1-treated nuclei showed increased nuclear acetyl-CoA generation, 2.5-fold over that of vehicle-treated controls (Fig. 5B). For comparison, we measured de novo acetyl-CoA synthesis in isolated mitochondria, and, consistent with our whole-cell data, PDC activity was reduced to one-fifth of control levels in mitochondria isolates from TGF-β1-treated cells (Fig. 5B). However, mitochondrial PDC activity remained at least two-fold higher than nuclear activity, suggesting that the majority of PDC activity is mitochondrial irrespective of TGF-β1 exposure.

### TGF-β1 decreases global protein acetylation by a PDK/PDC-dependent mechanism

**Figure 6:**
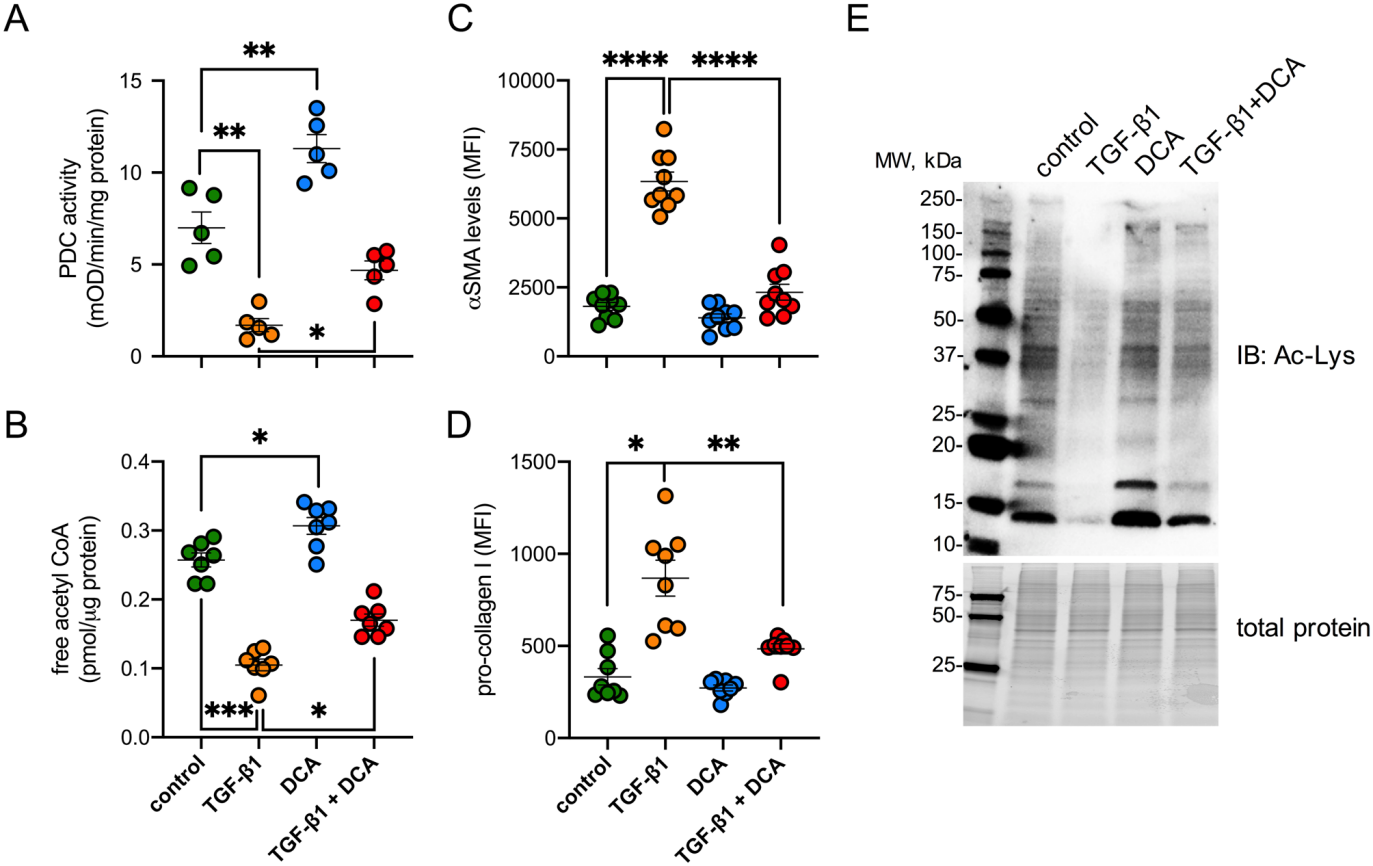
Inhibition of PDK with dichloroacetate (DCA) ameliorates the suppressive effect of TGF-β1 on PDC activity, acetyl-CoA levels and protein acetylation in UUOF. (**A**, **B**) the effect of TGF-β1 (1 ng/mL), DCA (5 mM) or co-treatment on (**A**) PDH activity, (**B**) free acetyl-CoA concentrations, (**C**) α-SMA levels and (**D**) pro-collagen I in UUOF. Measurements in (**A**, **B**) were normalised to total protein content. (**A–D**) Plots show mean ± SEM with pooled data from 2–3 independent experiments. *P < 0.05; **P < 0.01; ***P < 0.001; ****P < 0.0001. P-values were determined using Brown-Forsyth and Welch one-way ANOVA with Dunnett’s T3 multiple comparisons test. (**E**) Is representative of similar findings from 4 independent experiments probed in parallel.

Our working hypothesis was that changes in acetyl-CoA biosynthesis would result in global changes in protein acetylation. To test this we examined the effect of sodium dichloroacetate (DCA), a small molecule inhibitor of PDK1 and therefore PDC phosphorylation^17^. Consistent with its mode of action, DCA enhanced PDC activity (Fig. 6A) and free acetyl-CoA levels (Fig. 6B). When added in combination with TGF-β1, DCA ameliorated the effect of TGF-β1 on PDC activity (Fig. 6A) and acetyl-CoA concentrations (Fig. 6B), while markedly diminishing the effect of TGF-β1 on αSMA expression (Fig. 6C) and pro-collagen I (Fig. 6D).

Considering that free acetyl-CoA availability is a determinant of protein acetylation^16^, we next examined changes in lysine acetylation at the whole cell level, in response to TGF-β1 and DCA, or in combination. Immunoblotting showed that TGF-β1 decreased global lysine acetylation with a reduction in multiple bands (Fig. 6E). These included bands at approximately 11 and 17 kDa consistent with reductions in histone H3 and H4 acetylation respectively, which we confirmed by immunoblotting using pan-specific antisera (Supplementary Fig. S5). DCA on its own appeared to enhance acetylation of some proteins compared to control, consistent with enhanced acetyl-CoA generation (Fig. 6B) and abrogated the effects of TGF-β1 when added in combination (Fig. 6C,D).

### Discussion

A defining characteristic of CKD, like other fibrotic pathologies, is the persistent activation of renal fibroblasts^1^. Our recent work has demonstrated that TGF-β1 induces a shift in metabolism from oxidative respiration to aerobic glycolysis in renal fibroblasts which is associated with functional activation^16^. In this study, we have expanded upon these observations to show that this metabolic reprogramming specifically involves the inactivation of the PDC through phosphorylation of Ser232, which is accompanied by a reduction in intracellular free acetyl-CoA levels and a concomitant decrease in global protein acetylation. Furthermore, we have found that the metabolic effect of TGF-β1 is specific to fibroblasts derived from injured kidneys, suggesting that these cells are primed to respond in this way by exposure to the injurious environment.

Although it has long been recognized that alterations in cellular metabolism play an essential role in helping tumour cells acquire and maintain their malignant properties, we are starting to appreciate that cellular metabolism is altered in many forms of disease. Of particular interest is the observation that in various organs, fibroblasts undergo dramatic metabolic reprogramming during activation to facilitate growth, proliferation and their synthetic activities^6,7,9,10,35^. In some respects this resembles the so-called Warburg effect seen in tumours, where there is a shift from oxidative phosphorylation to aerobic glycolysis to support hyper-proliferation and protein synthesis^4^.

Our analysis of the canonical pathways differentially regulated by TGF-β1, identified inhibition of acetyl-CoA biosynthesis by PDC as a potential metabolic switch regulating fibroblast activation (Fig. 1). The PDC catalyses a series of rate-limiting reactions for the oxidative decarboxylation of pyruvate to form acetyl-CoA. In higher eukaryotes, this complex comprises multiple heterotetramers of E1α and E1β surrounding a core of multiple copies of E2, E3 and E3BP subunits, in addition to homodimers of PDKs and heterodimers of PDPs creating an approximately 9 MDa multienzyme assembly. PDKs and PDPs exhibit unique tissue expression patterns and kinetic properties, and sensitivities to regulatory molecules^17^. The 78% reduction in PDC activity we have seen here (Fig. 2) is disproportionately greater than the 38% reduction in protein levels (Fig. 1), implying that PDC is predominantly regulated post-transcriptionally by TGF-β1 as with many other regulatory inputs to this complex. PDC activity is tightly controlled via specific kinases (PDK1-4) and phosphatases (PDP1-2) that regulate a reversible inactivating phosphorylation^17^. The divergent changes in PDK1 and PDP1 observed here in response to TGF-β1, are consistent with the increase in inactive PDH-E1α, which appears preferentially phosphorylated at Ser232 (Fig. 2). These findings were corroborated by over-expression of a mutant PDH-E1α, resistant to phosphorylation at Ser232, which ameliorated the inhibitory effect of TGF-β1 on PDC activity (Fig. 2).

Intriguingly, however, effects on PDC biosynthetic pathways were only observed in fibroblasts propagated from obstructed kidneys (Fig. 1). This builds on other recent work where we showed that UUOF have upregulated responses to cytokine stimulation compared to their normal uninjured counterparts^36,37^, suggesting that injury primes their response. The mechanism for this remains uncertain although fibroblasts from injured kidneys express more type II and type I TGF receptors than their normal counterparts^37^, while a reporter assay indicated an exacerbation of SMAD signalling^36^. Autocrine cytokine signalling loops are also apparent^36^. Findings here therefore further reinforce this notion, showing that such ‘priming’ is a generalised functional change involving not only the response to exogenous cytokines but also canonical metabolic pathways.

A serendipitous finding was that the loss of cytoplasmic PDH-E1α staining was paralleled by an increase in nuclear PDC (Fig. 4). The presence of PDP-1 but absence of PDK1 in the nucleus implies that nuclear and mitochondrial PDH are regulated quite differently. Elegant studies from Sutendra et al.^32^ first demonstrated that PDC was not exclusively confined to mitochondria, but could also be found in the nucleus in response to environmental cues. Likewise various enzymes and components of the PDC have now been localised, at least transiently, in the nucleus in other situations including the mouse embryo^38^, nasopharyngeal^39^ and prostate^33^ carcinomas. This is however, to our knowledge, the first demonstration that TGF-β1 is a causative factor. In our case we are unable to say if this was a de novo assembly of the PDC, or as suggested by Sutendra et al.^32^, a translocation across the nuclear membrane.

An increase in acetyl-CoA-generating nuclear PDC does not however seem consistent with our other recent observations in renal fibroblasts that TGF-β1 induces a reduction in the both nuclear acetyl-CoA and histone acetylation^16^. One possibility is that nuclear PDC might be fulfilling other ‘moonlighting’ functions unrelated to acetyl-CoA generation. Interestingly, our ultrastructural studies reveal that nuclear PDC is localised to the nucleolus in TGF-β1-treated fibroblasts (Fig. 4). Clearly we cannot discount a role in histone acetylation, but we do note that the nucleolus contains more than 700 different proteins^40^, many involved in rRNA synthesis, and a specific role in non-histone protein acetylation is possible if acetyl-CoA synthesis by PDC were to be spatially coupled to nucleolar function. Even with respect to histone acetylation, our previous findings demonstrated that while there was a net decrease in histone acetylation (also shown here in Supplementary Fig. S5), changes at the individual mark were highly variable with acetylation of various marks being increased (H3K18, H3K27) or decreased (H3K4, H3K9, H3K14, H3K23) to differing degrees^16^. Nevertheless, this does serve to emphasise the compartmentalisation of PDC and its regulation, which may help account for divergent changes in acetylation of individual proteins in different subcellular, even sub-organelle, locales as a local source of acetyl-CoA. Although our results imply that the nuclear complex is present and active (Fig. 5), the mass spectrometry data highlighted that mitochondria retain greater acetyl-CoA biosynthetic capacity than the nucleus in TGF-β1-treated fibroblasts, despite markedly suppressed mitochondrial PDC activity.

Not only is acetyl-CoA a central metabolite that interconnects multiple metabolic pathways^41^, it is also a substrate for protein acetylation^22^. Nuclear histone acetylation has long been recognised as a major regulator of gene expression through chromatin reorganisation^42^. Recent studies have shown that the accumulation of PDC modulates acetylation in a large number of cellular proteins with multiple acetylation sites, suggesting that the role of acetylation extends well beyond histones^21,43^. Acetylation controls a plethora of regulatory processes, at multiple levels, from transcription right through to protein degradation^44^. Acetylated lysine residues change both size and electrostatic charge of amino acid side chains, alter enzymatic activity through binding preference, interact with other modifications competitively and create new protein docking sites^44^. These reactions can occur in multiple compartments, including the nucleus, cytoplasm, and mitochondria. Interestingly, the acetylation of mitochondrial proteins, including PDC itself^43,45,46^, implies a possible substrate-level feedback mechanism. Accordingly, in conjunction with the TGF-β1-induced reduction in acetyl-CoA, we were able to show that TGF-β1 produced a commensurate decrease in lysine acetylation of many different proteins (Fig. 6), thus extending our earlier findings relating to the suppressive effect of TGF-β1 on histone H3 acetylation^16^. The effect on protein acetylation was ameliorated by DCA (Fig. 6), a small molecule inhibitor of PDK, which maintains PDC in an unphosphorylated catalytically active form^47^. Taken together these novel observations serve to substantiate a link between PDC regulation, acetyl-CoA availability and global protein acetylation. Indeed, the fact that protein acetylation represents one of the most common post-translational modifications in cell biology^48^, implicates a more global integration of metabolic and protein acetylation reprogramming in fibrosis.

A growing body of evidence attests to the potential significance of these metabolic adaptations in vivo. Interrogation of publicly available datasets reveal that PDHA1 protein levels are decreased in folic acid and UUO murine models of kidney disease^49^, while phosphorylated Ser232 levels increase during early glomerular injury in Dahl salt-sensitive hypertensive rats^50^ . With respect to transcriptome analyses, *PDHA1* mRNA levels are significantly decreased during spontaneous kidney disease progression in Col4a3^*−/−*^ mice^51^ and mRNA levels are inversely associated with eGFR in patients with diabetic or IgA nephropathy in human CKD (Nephroseq v5, University of Michigan, Ann Arbour, MI, USA).

Emerging preclinical evidence suggests that interfering with specific metabolic pathways may be effective in the treatment of many diseases, with therapeutic approaches targeting metabolites in cancer^52^, heart disease^53^ and neurogenic disorders^54^ being actively proposed. In the context of this study, specific targets include enzymes responsible for glycolytic flux and pyruvate production^6^. While in vivo studies in fibrosis are lacking, parallel approaches elsewhere highlight that this may be a rational treatment strategy in renal fibrosis and fibrosis in general.

There are a number of limitations to these studies. Our experiments have only examined a single time point and dose of TGF-β1. Furthermore, our examination of global lysine acetylation has not characterised individual proteins. Time course studies will be needed to examine the temporal effects of TGF-β1 and the kinetics of acetylation^55^, while an unbiased detailed assessment of the acetylome^56^ with TGF-β1 treatment will be key to understanding the functional significance of this metabolic regulation. Finally, the relevance of these findings remains to be confirmed in vivo.

Notwithstanding these limitations, the observation that TGF-β1 induces profound changes in cellular metabolism during renal fibroblast activation, in part via regulatory effects centred on PDC, provides new insight into our understanding of TGF-β1 signalling and an opportunity to develop new approaches for preventing fibrosis in CKD. The important future objective is to confirm the in vivo significance of these metabolic dependencies and establish their clinical relevance.

## Materials and methods

### Cell culture and treatments

Primary banked cell cultures of fibroblasts propagated from fibrotic kidneys (3 days after UUO) (UUOF) and normal kidneys (NRKF) of male Sprague–Dawley rats were utilised for these studies^57^. The relevant procedures were approved by the Royal Melbourne Hospital Institutional Animal Ethics Committee. All experiments were conducted in accordance with the *Australian Code of Practice for the Care and Use of Laboratory Animals for Scientific Purposes*. Cultures were maintained in Dulbecco’s modified Eagle Medium (DMEM; Sigma, St. Louis, MO, USA) supplemented with 10% foetal bovine serum (FBS; Biocore, Melbourne, Vic, Australia), 2.2% HEPES, 1% l-glutamine, penicillin (50 U/mL) and streptomycin (50 μg/mL) (all Sigma) in a humidified incubator at 37˚C and 5% CO_2_. For experimental work, cells were seeded into 6-well plates (Costar, Corning, NY, USA) at 1 × 10^6^ cells/well for Western blotting or in 25 cm^2^ flasks (Costar) at 5 × 10^6^ cells/flask for flow cytometric or metabolic analyses. After attachment overnight and removal of floating cells, fibroblasts were typically cultured for a further 24 h in maintenance growth medium before switching to FBS-reduced media (1% FBS) for 24 h before experiments.

Fibroblasts were treated with 1 ng/mL recombinant human TGF-β1 (Sigma cat# T7039) in DMEM/1% FBS for 24 h. In the control groups, cells were treated with DMEM/1% FBS only. The dose of TGF-β1 administered was based on that previously used to stimulate αSMA expression in (myo)fibroblasts^16^. Additional incubations were performed with or without 5 mM DCA (Sigma cat# 347,795) dissolved in deionised water^47^ and 100 µM CPI-613 dissolved in DMSO (Sigma cat**#** SML0404)^58^.

### Canonical pathway analysis

Canonical pathway analysis of our previously published RNA-seq dataset^16^ (https://doi.org/10.6084/m9.figshare.7464818) was performed using the Ingenuity Pathway Analysis platform (IPA; Ingenuity Systems, Redwood City, CA, USA). IPA uses a literature-based knowledge base of known interactions between transcriptional regulators and their target genes to define an expected expression pattern of targets within each pathway for activated (Z-score ≥ 2) or inhibited (Z-score ≤ − 2) states. The P-value provides a measure of how likely the observed association between a specific pathway and each dataset would be if due to chance alone. Core analysis was performed using default settings: direct and indirect relationships supported by experimentally observed data in rat studies contained within the Ingenuity Knowledge Base. Network scores were calculated using a hypergeometric distribution and right-tailed Fisher's exact test and derived by IPA based on the number of targets and their proportion of the total dataset.

### Flow cytometry

Staining for unconjugated antisera was performed using standard flow cytometry protocols for indirect detection as described elsewhere^59^. The primary antibodies used were mouse anti-PDH-E1α (Abcam, Cambridge, UK; cat# ab110334) and mouse anti-PDK1 (cat# ab110025) and rabbit anti-pro-collagen I (Merck: cat# ABT257). A direct staining protocol was used for intracellular αSMA staining as previously described, using an Alexa Fluor 647 rabbit monoclonal antibody (Abcam: cat# ab196919)^59^. In each instance a minimum of 10,000 cells were examined on a BD FACSVerse (BD Biosciences, San Jose, CA, USA). Results were analysed using FlowJo LLC (Ashland, OR, USA) and are reported as mean fluorescent intensity (MFI).

### Immunocytochemistry

As described previously^59^, fibroblasts were seeded onto glass coverslips (Carl Zeiss, Oberkochen, Germany) in 6 well plates at low density (1 × 10^5^/well), and treated for 24 h as described. After treatment, cells were incubated with 100 nM MitoTracker Deep Red (Molecular Probes, Eugene, OR, USA) for 4 h at 37 °C before coverslips were washed, fixed and permeabilised in 4% paraformaldehyde containing 0.2% TritonX-100, washed again and post-fixed in 4% paraformaldehyde in PBS for 5 min. Cells were then blocked in 10% normal goat serum/3% BSA/0.1 M glycine in PBS before incubation with mouse anti-PDH-E1α (clone 8d10e6; Abcam cat# ab110334) diluted 1:100 in blocking buffer for 2 h at RT in a humidified chamber. Coverslips were then washed three times in PBS (5 min each) and incubated with Alexa Fluor 488-conjugated goat anti-mouse IgG (Life Technologies, Carlsbad, CA, USA; cat# A11001). In co-labelling experiments sections were incubated in parallel with anti-fibrillarin directly conjugated to Alex Fluor 594 (Abcam: cat# ab203400). Nuclei were stained with DAPI (1 μg/mL diluted in PBS) for 15 min at RT, before coverslips were washed and mounted in Vectashield HardSet (Vector Laboratories, Burlingame, CA, USA) and the edges sealed with nail varnish. Images were acquired the same day using a Leica SP5 confocal microscope with a 63 × oil immersion objective. High power images of individual nuclei were taken at 4 × optical zoom, yielding a resolution of 70 pixels per micron, permitting unambiguous visualization of PDC in the mid-plane of the nucleus. Each channel was acquired sequentially eliminating overlap between emissions of the secondary antibody conjugate and nuclear/mitochondrial stains. Fiji Image J V1.52b software^60^ was used to calculate representative intensity plots of PDH-E1α and DAPI signals along a midplane line using the plot profile function.

As previously described by us^59^, 3D rendered volumes were created from 30 × 0.25 µm optical sections. Image stacks were directly imported into Huygens Professional v19.04 (Scientific Volume Imaging, Hilversum, The Netherlands; https://svi.nl) for deconvolution and volume rendering. All high magnification images were deconvoluted using a theoretical point spread function for each channel, and the classical likelihood estimation algorithm. The signal to noise ratios and background intensities were automatically determined and taken into account during processing.

### Isolation of nuclei and mitochondria

Isolation of nuclei or mitochondria was performed using commercially available kits (Nuclei Isolation Kit: Nuclei PURE Prep; Sigma; cat# NUC201 and Mitochondria Isolation Kit, Sigma; cat# MITOISO2) according to the manufacturer’s instructions. Crude mitochondrial isolates were further purified by Percoll density gradient centrifugation. Purity was confirmed by immunoblotting and/or confocal microscopy. Freshly prepared isolates were used for all functional measurements. For Western blotting, isolated nuclei and mitochondria were lysed on ice for 30 min in ice-cold RIPA containing Halt Protease and Phosphatase Inhibitor Cocktail (Thermo cat# 78,440) with periodic vortexing. Soluble fractions were obtained by centrifugation at 10,000×*g* for 10 min and the total protein content quantified by BCA assay (Bio-Rad). Aliquots were stored at − 80 °C until analysis.

### Western blotting

Western blotting studies were performed as previously described^61^. Equal amounts of heat-denatured proteins (50 µg) were resolved on Any kD Mini-PROTEAN TGX stain-free pre-cast gels (Bio-Rad, Hercules, CA, USA) under reducing conditions, transferred onto PVDF with the Trans-Blot Turbo transfer system (Bio-Rad) and blocked in either 5% (wt/vol) non-fat milk or BSA in TBS containing 0.1% Tween-20 (TBST) for 1 h at RT. Membranes were variously probed with polyclonal rabbit anti-acetylated lysine (Cell Signalling Technology, Danvers, MA, USA; cat # 9441), rabbit monoclonal anti-PDH-E1α (clone EPR11098; Abcam: cat# ab168379), anti-pSer232 PDH-E1α (Merck Millipore: cat# AP1063), anti-pSer293 PDH-E1α (Merck Millipore: cat# AP1062), anti-PDK1 (Abcam: cat# ab110025), rabbit monoclonal anti-PGK1 (Abcam: cat# ab199438), rabbit polyclonal anti-PDP1 (Abcam: cat# ab198261), rabbit monoclonal anti-Tomm20 (Abcam: cat# ab186735), mouse monoclonal anti-CytC (BD Biosciences: cat# 556433), rabbit polyclonal anti-Lamin B1 (Abcam: cat# ab16048), rabbit polyclonal pan-acetyl histone H3 (Active Motif: cat# 61637), rabbit polyclonal pan-acetyl histone H4 (Active Motif: cat# 39926), rabbit polyclonal anti-histone H3 (Cell Signalling Technology: cat# 9715S) or rabbit polyclonal anti-histone H4 (Cell Signalling Technology: cat# 2592S) overnight at 4 °C diluted in blocking buffer. Membranes were then incubated with an appropriate highly cross-adsorbed goat anti-rabbit IgG HRP-conjugated secondary (Invitrogen: cat # A16110) or goat anti-mouse IgG HRP-conjugated secondary (Invitrogen: cat # A16078) in blocking buffer for 2 h at RT. Blots were developed in Clarity Western ECL substrate (Bio-Rad) and imaged using the ChemiDoc Imaging System (Bio-Rad) running Image Lab software (Bio-Rad). A stain-free imaging workflow (Bio-Rad) was used to confirm equal protein loading and for normalisation^62^.

### Pyruvate dehydrogenase complex activity

The PDH Enzyme Activity Microplate Assay (Abcam: cat# ab109902;) was used to assess the ability of immunocaptured pyruvate dehydrogenase to convert pyruvate to acetyl-CoA by following the reduction of NAD^+^ to NADH, coupled to the reduction in a reporter dye. In brief, cells were trypsinised, pelleted and snap frozen in liquid nitrogen, before being stored at − 80 °C for batch analysis. Cell pellets were thawed, lysed with detergent, loaded onto assay plate and incubated at RT. After 3 h, wells were emptied, rinsed with stabilisation buffer, and assay solution was added. Absorbance was monitored at 450 nm for 15 min using a Synergy HTX mulitmode plate reader running Gen 5 data analysis software (BioTek, Winooski, VT, USA). Activity was expressed as a rate of reaction (ΔmOD_450_/min) normalised for total protein content determined using a Micro BCA Protein assay kit (Thermo Scientific: cat#23235).

### Quantitative RT-PCR

RNA was extracted using the Qiagen miRNeasy Mini kit and reverse-transcribed using the iScript RT supermix kit (Bio-Rad) as previously described^36^. Quantitative real-time PCR (qRT-PCR) was performed according to manufacturer’s instructions in a CFX96 cycler (Bio-Rad) using the SsoAdvanced Universal SYBR Green Supermix (Bio-Rad) using the following rat primer pairs (Sigma):

PDK1 5′ TCGAAAACACATTGGAAGCA-3′; 5′-GTCCTGGTGATTTCGCATTT-3′; PDK2 5′-AGGAAGTCAATGCCACCAAC-3′; 5′-TTTTGATGGGAGGGAGAGTG-3′; PDK3 5′-GTGGAGTCCCACTTCGAAAA-3′; 5′-ATTGGCAAGCCATAACCAAA-3′; PDK4 5′-CGTCGTCTTGGGAAAAGAAG-3′; 5′-CGTGAATTGTCCATCACAGG-3′.

The mRNA level of target genes was normalised to the reference gene, GAPDH, and expressed relative to vehicle-treated controls using the 2^(−DΔCt)^ method.

### Plasmid transfection

TGF-β1-treated UUOF were transiently transfected with pcDNA3.1 + /C-DYK expression plasmids (GenScript, Piscataway, NJ, USA) encoding full-length wild type rat PDH-E1α or substitution mutants S232A, S232D generated using the QuikChange site-directed mutagenesis kit (Agilent, Santa Clara, CA, USA). Plasmid DNA was titrated to optimize over-expression to the described level (Supplementary Fig. S6A). For experimental studies, cells were transfected with 5 μg of plasmid DNA (per 10^6^ cells) using the Viromer yellow reagent (Lipocalyx, Halle, Germany) according to the manufacturer’s protocol. Cells were ~ 60–70% confluent at the time of transfection. Transfection efficiency was determined by flow cytometry 24 h after transfection with pcDNA3.1 + C-eGFP vectors encoding the same inserts and was 70–85% (Supplementary Fig. S6B). Over-expression of wild-type or mutant PDH-E1α was confirmed by flow cytometry using mouse anti-PDH-E1α antisera with 5 µg plasmid. All treatments were performed in triplicate alongside cells treated with empty vector or transfection media only as controls.

### Multiplex assay

Simultaneous quantitation of total PDH, phosphorylated Ser232 PDH, phosphorylated Ser293 and phosphorylated Ser300 in cell lysates (10 µg/well) was performed using the Milliplex MAP Multi-species PDH complex magnetic bead panel (EMD Millipore, Burlington MA, USA, #PDHMAG-13 K) according to the manufacturer’s instructions.

### Acetyl-CoA metabolite assay

Intracellular levels of acetyl-CoA in whole-cell lysates were measured fluorometrically (Ex/Em 535/587 nm) using the PicoProbe Acetyl CoA Assay Kit (Abcam: cat #ab87546), according to the manufacturer’s instructions as previously described and Synergy HTX multimode plate reader (BioTek)^16^. In brief, cells were harvested and stored at − 80 °C until batched analysis. For whole-cell analysis, cell pellets were homogenised in assay buffer, clarified by centrifugation (10,000×*g* for 10 min at 4 °C) and then deproteinised in 1 M perchloric acid with the excess removed and neutralised by adding 2 M KOH (pH 7.0 ± 0.5). Concentrations of free acetyl-CoA (pmol) were calculated from a standard curve and normalised for the protein concentration (BCA).

### Mass spectrometry for isotope-labelled acetyl-CoA

The experimental protocol for mass spectrometry of ^13^C_1_-acetyl-CoA was based on the method of Sutendra et al.^32^. Briefly, immediately following isolation, nuclei (1–2 × 10^6^/mL) or mitochondria (1–2 mg protein/mL) from control or TGF-β1-treated UUOF, were incubated with ^13^C_2_-pyruvate (Sigma-: cat# 490,725) at the stated concentration for 10 h at 37 °C in storage buffer, before the reaction was terminated with the addition of ice-cold storage buffer. Nuclei/mitochondria were pelleted by centrifugation and washed in buffer to remove excess pyruvate. Pellets were resuspended in ice-cold 80% methanol and vigorously vortexed for 5 min before snap freezing in liquid N_2_. Samples were thawed on ice and the freeze–thaw cycle repeated twice before the debris was pelleted at 20,000×*g* for 10 min at 4 °C and the supernatants dried in a speed vacuum concentrator. Extracts were reconstituted in sample solvent and analysed by LC/MS as described above.

### Statistical analysis

Data are expressed as individual data points and mean. GraphPad Prism v8.4.2 (GraphPad, San Diego, CA, USA) was used to plot data and for statistical analysis. Results were analyzed using Welch’s t test, Brown-Forsyth and Welch one-way ANOVA with Dunnett’s T3 multiple comparisons test or two-way ANOVA with Sidak’s multiple comparisons test as appropriate. Two-tailed P-values < 0.05 were considered statistically significant.

## Supplementary informationSupplementary information

Supplementary Information.
