# Supplementary material for: TGF-β1 is a regulator of the pyruvate dehydrogenase complex in fibroblasts
Source: Sci Rep. 2020 Oct 21;10:17914. doi: 10.1038/s41598-020-74919-8 (PMC7578649; doi:10.1038/s41598-020-74919-8)
Supplement: Supplementary file 1 — Supplementary Information. [file 41598_2020_74919_MOESM1_ESM.pdf]

Supplementary material

for

TGF-β1 is a regulator of the pyruvate dehydrogenase complex in fibroblasts

Edward R. Smith[1,2], Timothy D. Hewitson[1,2]

[1]Department of Nephrology, The Royal Melbourne Hospital (RMH) and [2]Department of Medicine - RMH, University of Melbourne, Australia.

Address for correspondence:
Timothy D. Hewitson
Department of Nephrology
The Royal Melbourne Hospital
Grattan Street,
Parkville, Victoria 3050, Australia
Email: tim.hewitson@mh.org.au

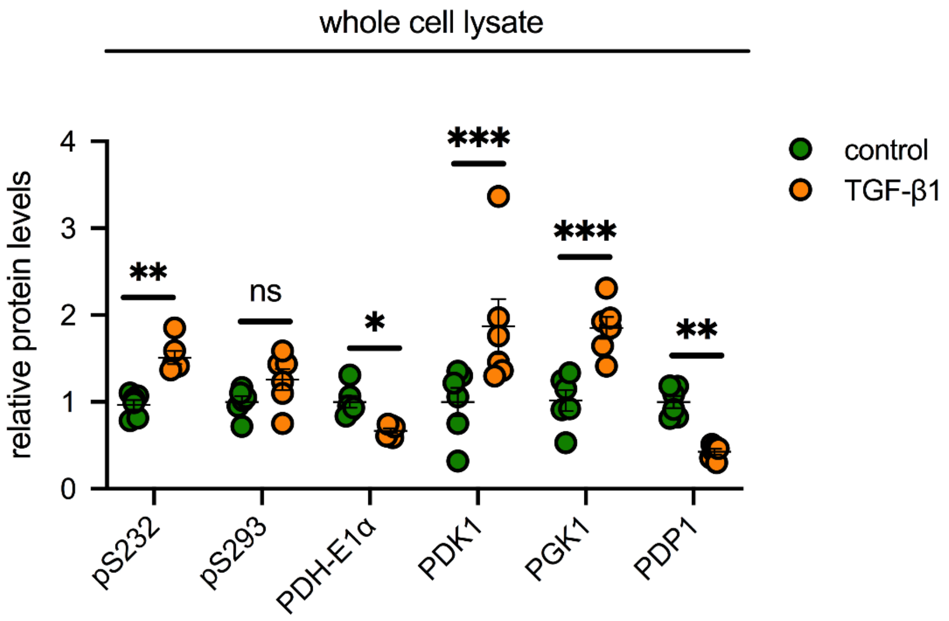

Supplementary Figure S1. Quantification of phosphoserine 293 (pS232), phosphoserine 232 (pS293) and total PDH-E1α, PDK1, PGK1 and PDP1 protein levels in whole cell lysates of UUOF treated with vehicle or 1 ng/mL TGF-β1 for 24h. Band intensities were normalised to total protein using the stain-free workflow and expressed relative to control levels (mean=1). Plot shows mean ± SEM of 6 replicates from 3 independent biological experiments. *P<0.05; **P<0.01; ***P < 0.001. P-values were determined using unpaired t tests and the two-stage method of Benjamini, Krieger and Yekutieli (FDR=1%).

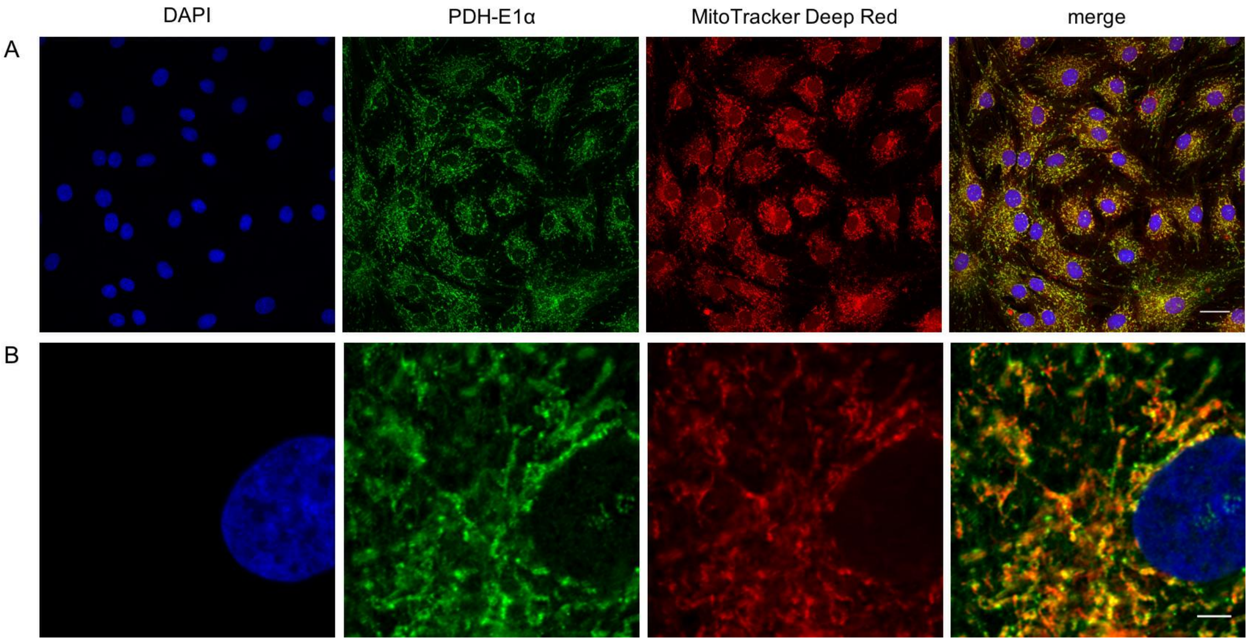

Supplementary Figure S2. Localisation of PDH-E1α to mitochondria in control UUOF. Representative low-power (A) and high-power (B) confocal imaging of PDC subunit PDH-E1α (green), the mitochondrial stain MitoTracker Deep Red (red) and DAPI (blue) in untreated fixed UUOF. Scale bar A = 20 μm; B = 3 μm

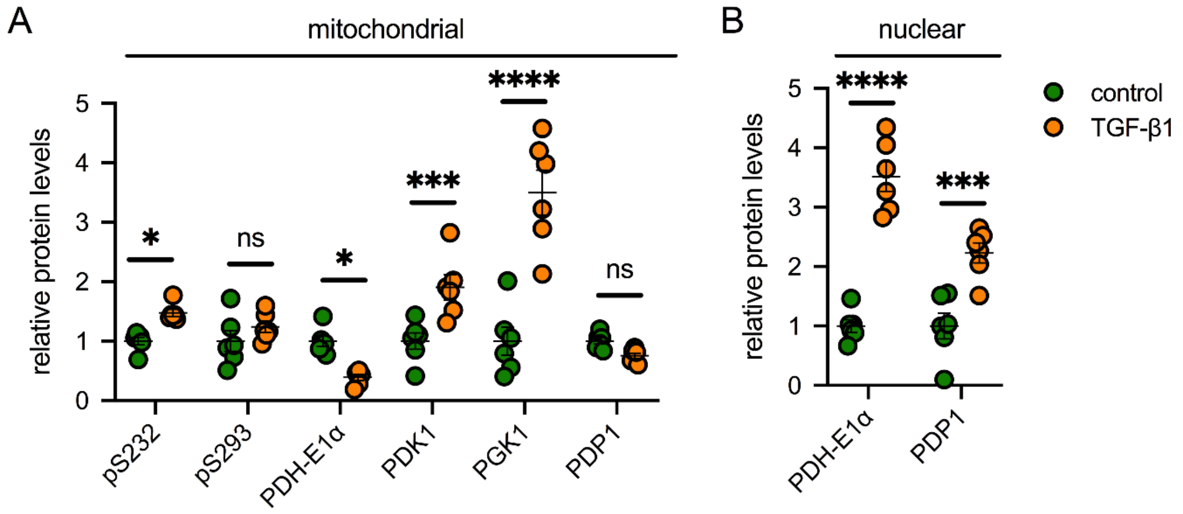

Supplementary Figure S3. Quantification of phosphoserine 293 (pS232), phosphoserine 232 (pS293) and total PDH-E1α, PDK1, PGK1 and PDP1 protein levels in **A** mitochondrial and **B** nuclear extracts of UUOF treated with vehicle or 1 ng/mL TGF-β1 for 24h. Band intensities were normalised to total protein using the stain-free workflow and expressed relative to control levels (mean=1). Plot shows mean ± SEM of 6 replicates from 3 independent biological experiments. *P<0.05; ***P<0.001; ****P<0.0001. P-values were determined using unpaired t tests and the two-stage method of Benjamini, Krieger and Yekutieli (FDR=1%).

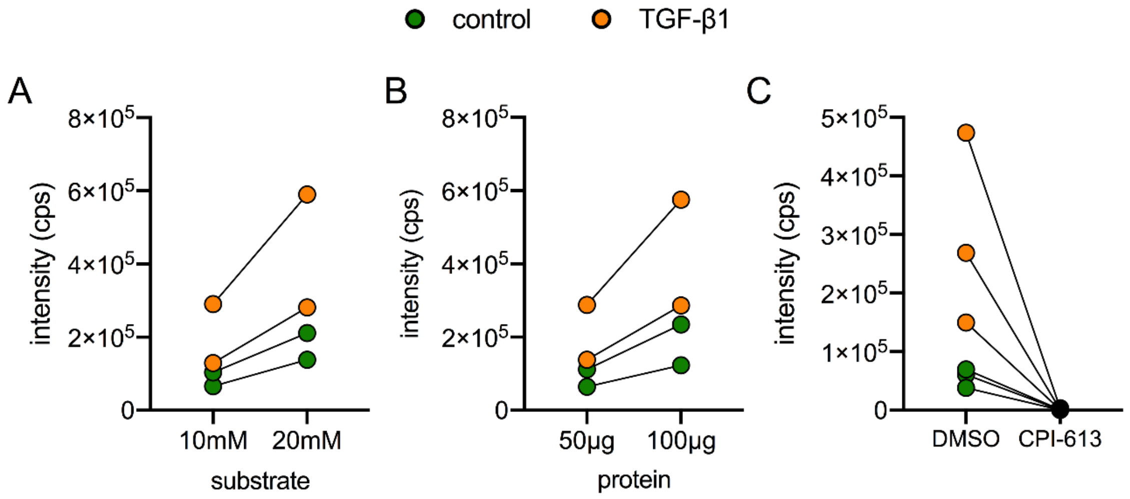

Supplementary Figure S4. Validation of MS assay for nuclear PDC activity. A-C, PDC activity in isolated nuclei with or without TGF-β1 (1ng/mL) pre-treatment was determined by LC-MS/MS (cps, counts per second). Freshly prepared isolates of TGF-β1 or vehicle-treated UUOF were incubated with $^{13}C_2$-pyruvate for 10h at 37°C after which metabolites were extracted for quantitation of $^{13}C_1$-acetyl CoA. Nuclear PDC activity increased proportionately with increasing amounts of substrate (**A**) and protein (**B**) and was completely inhibited by pre-treatment (**C**) with CPI-613 (150 µM, black circles) in both vehicle (green circles) and TGF-β1-treated (orange circles) cells (n=2 each condition).

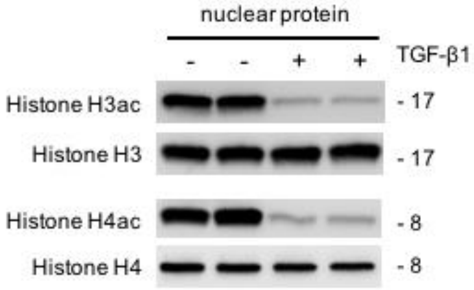

Supplementary Figure S5. Effect of TGF-β1 on histone H3 and H4 acetylation in UUOF. Western blots for acetyl-H3 and acetyl-H4 in nuclear protein extracts from control and TGF-β1-treated UUOF (1 ng/mL, 24h). Total histone H3 and H4 were probed to demonstrate equivalent loading. Representative blot of 3 independent experiments.

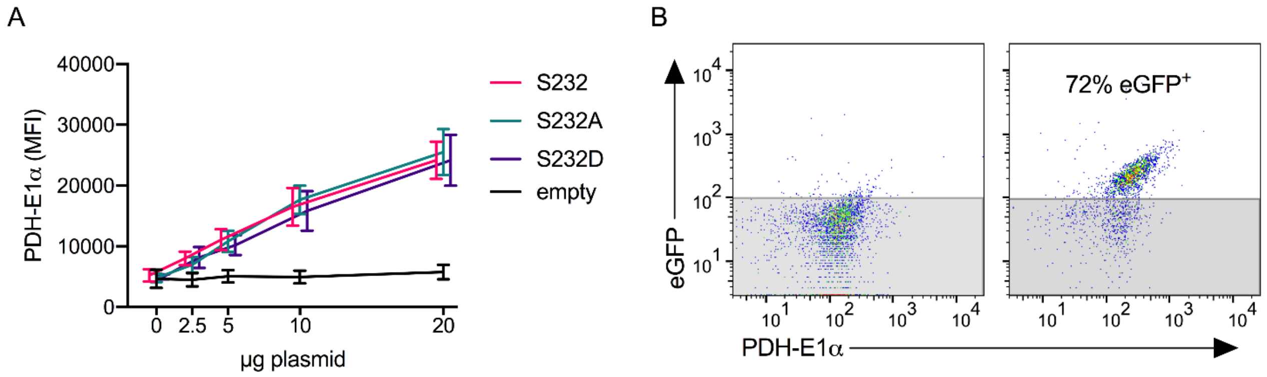

Supplementary Figure S6. Transfection of UUOF with PDH-Eα1 plasmids. A, UUOF were transiently transfected with increasing amounts (2.5 to 20 µg) of pcDNA3.1+/C-DYK expression plasmids full-length wild type rat PDH-E1α (S232) or substitution mutants S232A, S232D. PDH-E1α expression was confirmed by flow cytometry using mouse anti-PDH-E1α antisera. Transfection with empty vector was used as a control. Plot shows mean ± SEM mean fluorescence intensity of labelling. **B**, UUOF were transiently transfected with pcDNA3.1+ expression plasmids full-length wild type rat PDH-E1α (S232) fused with eGFP at the C-terminus (right). eGFP and PDH-E1α expression was analyzed by flow cytometry 24h after transfection. Cells were gated as shown in panel a discriminating between GFP− cells (grey shaded region) and cells expressing high levels of eGFP. Mean transfection efficiency was 70-85%.

Uncropped blots

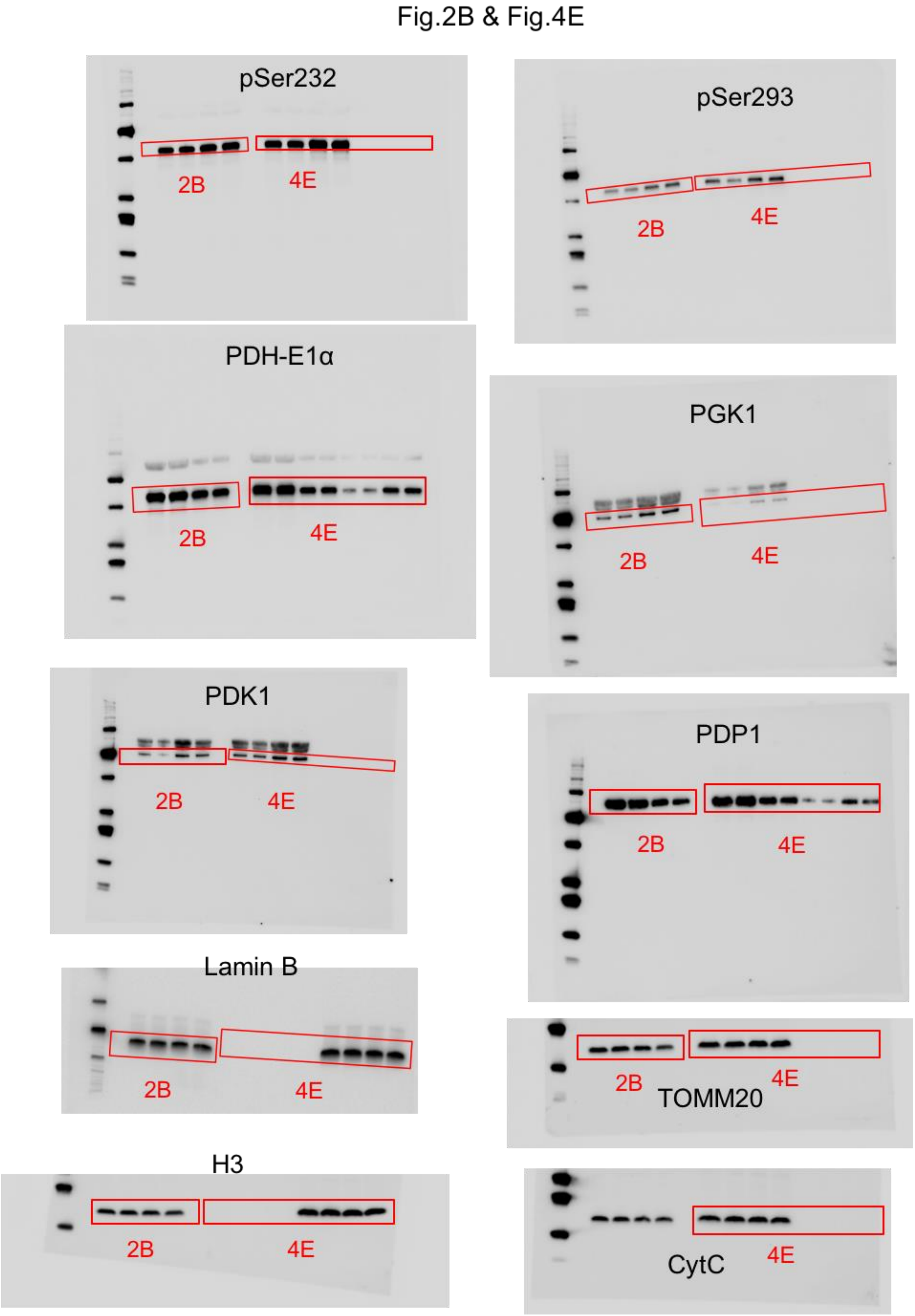

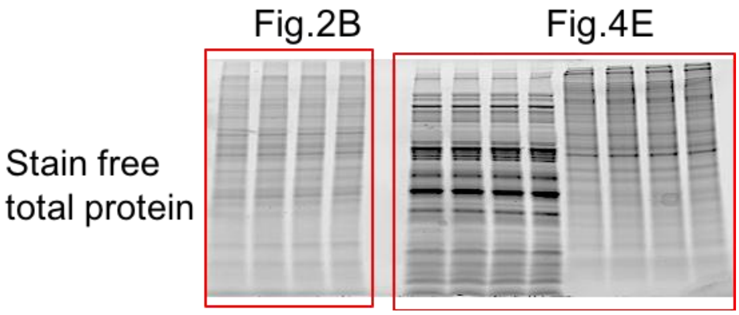

Fig.2B
Fig.4E
Stain free
total protein

Fig.5D

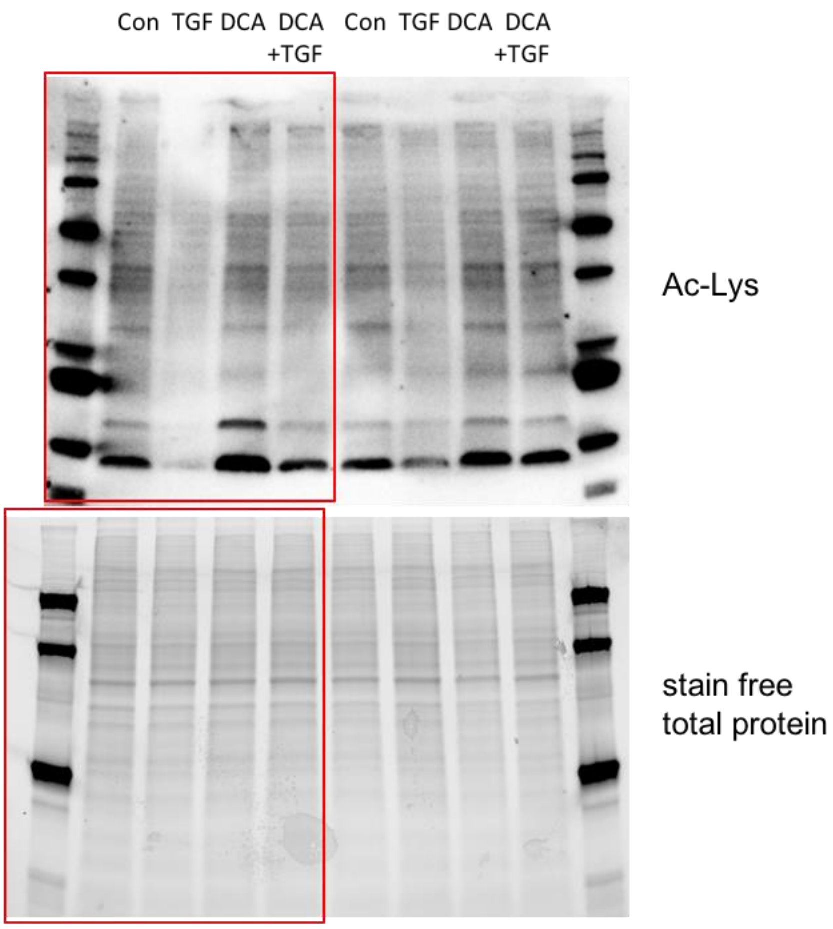
Con TGF DCA DCA +TGF Con TGF DCA DCA +TGF
Ac-Lys
stain free total protein

Fig.S4

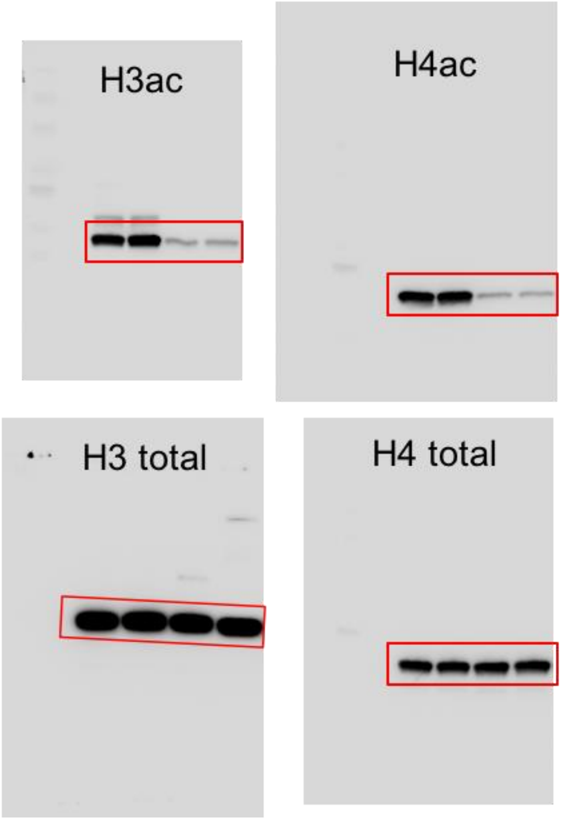
H3ac
H4ac
H3 total
H4 total
